# Prevalence of intestinal protozoa infection among school-aged children on Pemba Island, Tanzania, and effect of single-dose albendazole, nitazoxanide and albendazole-nitazoxanide

**DOI:** 10.1186/1756-3305-6-3

**Published:** 2013-01-04

**Authors:** Benjamin Speich, Hanspeter Marti, Shaali M Ame, Said M Ali, Isaac I Bogoch, Jürg Utzinger, Marco Albonico, Jennifer Keiser

**Affiliations:** 1 Department of Medical Parasitology and Infection Biology, Swiss Tropical and Public Health Institute, P.O. Box, CH-4002, Basel, Switzerland; 2 University of Basel, P.O. Box, CH-4003, Basel, Switzerland; 3 Department of Medical and Diagnostic Services, Swiss Tropical and Public Health Institute, P.O. Box, CH-4002, Basel, Switzerland; 4 Public Health Laboratory (Pemba)-Ivo de Carneri, P.O. Box, TZ-122, Wawi, Chake Chake, Tanzania; 5 Divisions of Internal Medicine and Infectious Diseases, Toronto General Hospital, Toronto, Ontario, Canada; 6 Department of Epidemiology and Public Health, Swiss Tropical and Public Health Institute, P.O. Box, CH-4002, Basel, Switzerland; 7 Ivo de Carneri Foundation, P.O. Box, IT-10122, Milan, Italy

**Keywords:** Intestinal protozoa, *Blastocystis hominis*, *Entamoeba histolytica/E. dispar*, *Giardia intestinalis*, Albendazole, Nitazoxanide, Ether-concentration method

## Abstract

**Background:**

Pathogenic intestinal protozoa infections are common in school-aged children in the developing world and they are frequently associated with malabsorption syndromes and gastrointestinal morbidity. Since diagnosis of these parasites is difficult, prevalence data on intestinal protozoa is scarce.

**Methods:**

We collected two stool samples from school-aged children on Pemba Island, Tanzania, as part of a randomized controlled trial before and 3 weeks after treatment with (i) single-dose albendazole (400 mg); (ii) single-dose nitazoxanide (1,000 mg); (iii) nitazoxanide-albendazole combination (1,000 mg–400 mg), with each drug given separately on two consecutive days; and (iv) placebo. Formalin-fixed stool samples were examined for the presence of intestinal protozoa using an ether-concentration method to determine the prevalence and estimate cure rates (CRs).

**Results:**

Almost half (48.7%) of the children were diagnosed with at least one of the (potentially) pathogenic protozoa *Giardia intestinalis*, *Entamoeba histolytica/E. dispar* and *Blastocystis hominis.* Observed CRs were high for all treatment arms, including placebo. Nitazoxanide showed a significant effect compared to placebo against the non-pathogenic protozoon *Entamoeba coli.*

**Conclusions:**

Intestinal protozoa infections might be of substantial health relevance even in settings where they are not considered as a health problem. Examination of a single stool sample with the ether-concentration method lacks sensitivity for the diagnosis of intestinal protozoa, and hence, care is indicated when interpreting prevalence estimates and treatment effects.

## Background

Infection with pathogenic intestinal protozoa (e.g. *Entamoeba histolytica* and *Giardia intestinalis*) result in considerable gastrointestinal morbidity, malnutrition and mortality worldwide, particularly among young children in developing countries [1,2]. It has been estimated that *E. histolytica*, the causative agent of amoebiasis, kills between 40,000 and 100,000 people each year; hence, is one of the deadliest parasitic infections worldwide [2,3]. In the People’s Republic of China alone, *G. intestinalis* affects an estimated 28.5 million people every year [1]. The prevalence of *G. intestinalis* has been estimated at 2–3% in the industrialized world and 20–30% in developing countries [4]. *Cryptosporidium* spp*.* is another major causal agent of diarrhoea, primarily affecting immunocompromised individuals such as those infected with HIV [3,5,6]. *Blastocystis hominis* is a common additional anaerobic intestinal protozoon and its pathogenicity is still under debate [7-9]. Lack of access to clean water, sanitation and hygiene are strong drivers for infection with intestinal protozoa [10-12].

Several drugs are currently available to treat intestinal protozoa infections. Most commonly used are 5-nitroimidazole compounds, including metronidazole, tinidazole, ornidazole and secnidazole [13]. Alternative effective agents, when given as multiple doses, include nitazoxanide and albendazole [14-16].

**Figure 1:**
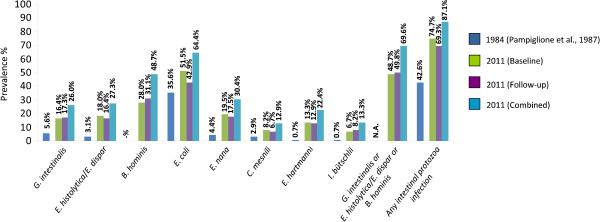
**Comparison of prevalence of intestinal protozoa infections assessed in mid-2011 at baseline, 3 weeks post-treatment follow-up and combined results (current study) against prevalence determined in 1984 (study done by Pampiglione*et al.*[**[17]**]) on Pemba Island, Tanzania.**

Information on the prevalence of intestinal protozoa infections is scarce and little data are available from sub-Saharan Africa. For example, to the best of our knowledge, the prevalence of intestinal protozoa infections on Pemba Island has been assessed only twice and these investigations date back to 1984 and 1992 [17,18]. In the 1984 study, the prevalence of *G. intestinalis* and *E. histolytica* among children and adults combined were 5.6% and 3.1%, respectively. Prevalences of 35.6%, 4.4%, 2.9%, 0.7% and 0.7% were reported for *Entamoeba coli*, *Endolimax nana*, *Chilomastix mesnili*, *Entamoeba hartmanni* and *Iodamoeba bütschlii,* respectively (Figure 1) [17]. The study conducted in 1992 reported prevalences of 25.4% for *E. histolytica* and 6.6% for *G. intestinalis* among children aged 9–17 years [18].

The study reported here was integrated in a randomized controlled trial carried out in school-aged children on Pemba Island, Tanzania in mid-2011 to asses the efficacy and safety of single-dose nitazoxanide and albendazole and a nitazoxanide-albendazole combination against *Trichuris trichiura* and other soil-transmitted helminth infections [19]. The aim of the present work was to evaluate the prevalence of intestinal protozoa infections in these children and to determine whether the study drugs had an effect on intestinal protozoa. Throughout the study, the ether-concentration method was used on formalin-fixed stool samples.

## Methods

### Ethics statement

Our randomized controlled trial was approved by the ethics committee of Basel (EKBB; reference no. 225/10), and the Ministry of Health and Social Welfare of Zanzibar (ZAMREC; reference no. 0001/010) and is registered at Current Controlled Trials (trial identifier: ISRCTN08336605). Participating in the trial required written informed consent from parents or legal guardians and oral assent from children. Parents and children were counseled that participation is voluntary and withdrawal was possible at any time without specification of reasons and further obligations. At the end of the trial, all children who were diagnosed with soil-transmitted helminths received albendazole (single oral dose of 400 mg).

### Study area and design

Details of the study area, population and procedures have been described previously [19]. In brief, the study was carried out in June and July 2011 in children aged 7–15 years attending two schools (Wawi and Al-Sadik) located within a radius of 10 km from Chake Chake, the main city of Pemba Island. The study was a randomized controlled trial with four treatment arms: (i) single-dose albendazole (400 mg); (ii) single-dose nitazoxanide (1,000 mg); (iii) nitazoxanide-albendazole combination (1,000 mg–400 mg) with each drug given separately on two consecutive days; and (iv) placebo. Of the 657 children, who completed the trial, 550 (83.7%) had formalin-fixed stool samples before and after treatment and were therefore included in the present study (treatment arms: (i) n = 136, (ii) n = 145, (iii) n = 117, and (iv) n = 152; Wawi school: n = 404, Al-Sadik school: n = 146).

### Procedures for the diagnosis of intestinal protozoa

Approximately 2 g of stool were fixed in 10 ml of 5% formalin in Falcon tubes labeled with unique identifiers. Stool samples were thoroughly broken down and homogenized with a wooden spatula. The formalin-fixed samples were transferred to the Swiss Tropical and Public Health Institute (Basel, Switzerland) and examined within 10 months.

The fixed stool samples were processed with an ether-concentration method [20,21]. Briefly, the homogenized stool sample was filtered through a medical gauze into a new tube and then centrifuged for 1 min at 500 *g*. The supernatant was discarded. To the remaining pellet, 7 ml of physiological NaCl-solution and 2–3 ml of diethyl ether was added. Tubes were shaken and centrifuged again for 3 min at 500 *g*. The upper three layers were discarded. The entire sediment was examined by experienced laboratory technicians under a microscope for soil-transmitted helminths at a magnification of 100×, and intestinal protozoa at a magnification of 400× or 500× using oil immersion. The current analysis focuses on intestinal protozoa, including the pathogenic intestinal protozoa *G. intestinalis* and *E. histolytica*/*E. dispar* (of note, these two *Entamoeba* species cannot be differentiated by microscopy [22]); the potentially pathogenic protozoon *B. hominis*, and the non-pathogenic protozoa *E. coli*, *E. hartmanni*, *E. nana*, *C. mesnili* and *I. bütschlii*[7-9,23]. *Cryptosporidium* spp. was not included, since it cannot easily be detected with the formalin-ether concentration technique, and would have required staining with the modified Ziehl-Neelsen method [24]. Infection intensities were classified as follows: (i) negative (no cysts or trophozoites in the entire sediment); (ii) light (one to five cysts or trophozoites per slide); (iii) moderate (one cyst or trophozoite per observation field at the 400× or 500× magnification); and (iv) heavy (more than one cyst or trophozoite per observation field) [21,25].

### Statistical analysis

Data were double-entered into an Excel file (Microsoft 2010) and cross-checked. Statistical analysis was performed using Stata version 10.1 (StataCorp; College Station, USA).

The prevalence of intestinal protozoa was determined before treatment (baseline) and at the 3-week post-treatment follow-up. Differences between prevalence at baseline and follow-up were assessed using *x*^2^ test. In the baseline prevalence analyses, the odds of being infected with a specific intestinal protozoon species for boys compared to girls and for children from Wawi compared to Al-Sadik school was calculated using logistic regression.

Cure rates (CRs) were estimated for each intestinal protozoon species for the different treatment arms as the percentage of positive children at baseline diagnosed negative after treatment. Differences in CRs among treatment arms were examined using logistic regressions. Reduction of infection intensity was assessed as the difference in infection intensities before and after treatment among all individuals (negative individuals before treatment were included in the analysis). Mean of differences in infection intensities was calculated for each treatment arm and for each intestinal protozoon species together with 95% confidence intervals (CIs). Decrease in infection intensity was assumed as significant when 95% CI was below 0.

## Results

### Prevalence and intensity of intestinal protozoa infection

Based on the analysis of one formalin-fixed stool sample per child at baseline, 74.7% of the children harboured at least one intestinal protozoa species. About half of the children (48.7%) were infected with at least one of the three (potentially) pathogenic intestinal protozoa. The prevalence of *E. histolytica/E. dispar* and *G. intestinalis* was 18.0% and 16.4%, respectively before treatment (Table 1). The potentially pathogenic intestinal protozoon *B. hominis* was diagnosed in 28.0% of the children. Prevalences for the other intestinal protozoa were 51.5% for *E. coli*, 19.5% for *E. nana*, 13.3% for *E. hartmanni*, 8.2% for *C. mesnili* and 6.7% for *I. bütschlii.* For the pathogenic intestinal protozoon *G. intestinalis*, 44.4%, 38.9% and 16.7% of the infections were classified as light, moderate and heavy, respectively. The infection intensities of other intestinal protozoa species are listed in Table 1.

**Table 1:** Baseline characteristics of included school-aged children on Pemba Island in mid-2011 with regard to intestinal protozoa infection

**Characteristic, intestinal protozoa**	**N (prevalence in %)**	**Girls (%)/boys (%)**	**Wawi (%)/Al-Sadik school (%)**
	**low (%)/moderate (%)/heavy (%)**		
No. of children tested	550	271/279	404/146
*G. intestinalis*	90 (16.4)	37 (13.7)/53 (19.0)	59 (14.6)/31 (21.1)
	40 (44.4)/35 (38.9)/15 (16.7)		
*E. histolytica/E. dispar*	99 (18.0)	53 (19.6)/46 (16.5)	77 (19.1)/22 (15.1)
	57 (57.6)/33 (33.3)/9 (9.1)		
*B. hominis*	154 (28.0)	73 (26.9)/81 (29.0)	103 (34.9)/51 (25.5)
	125 (81.2)/25 (16.2)/4 (2.6)		
*E. coli*	283 (51.5)	151 (55.7)/132 (47.3)	210 (52.0)/73 (50.0)
	101 (35.7)/104 (36.7)/78 (27.6)		
*E. nana*	107 (19.5)	58 (21.4)/49 (17.6)	71 (17.6)/36 (24.7)
	62 (57.9)/40 (37.4)/5 (4.7)		
*E. hartmanni*	73 (13.3)	38 (14.0)/35 (12.0)	52 (12.9)/21 (14.4)
	57 (78.1)/14 (19.2)/2 (2.7)		
*C. mesnili*	45 (8.2)	27 (6.5)/18 (10.0)	31 (7.7)/14 (9.6)
	21 (46.7)/18 (40.0)/6 (13.3)		
*I. bütschlii*	37 (6.7)	18 (6.6)/19 (6.8)	22 (5.4)/15 (10.3)
	31 (83.8)/5 (13.5)/1 (2.7)		
*G. intestinalis* or *E. histolytica/E. dispar* or *B. hominis*	268 (48.7)	135 (49.8)/133 (47.8)	187 (46.3)/81 (55.5)
Any intestinal protozoa	411 (74.4)	219 (80.8)/192 (68.8)	297 (73.5)/114 (78.1)

Examination of the follow-up stool samples revealed the following prevalences: 49.8% of children remained infected with at least one of the three (potentially) pathogenic intestinal protozoa and 69.3% of the children were found positive for at least one intestinal protozoa. The prevalence of *G. intestinalis*, *E. histolytica/E. dispar* and *B. hominis* at the 3-week post-treatment follow-up was 17.3%, 16.4% and 31.1%, respectively (Figure 1). Prevalence for the non-pathogenic intestinal protozoa species were 42.9% for *E. coli*, 17.5% for *E. nana*, 12.9% for *E. hartmanni*, 8.2% for *I. bütschlii* and 6.7% for *C. mesnili.* According to the *x*^2^ test, only *E. coli* (*p* = 0.004) and all intestinal protozoa combined (*p* = 0.046) showed significantly lower prevalence at follow-up compared to baseline.

The odds of being infected with any intestinal protozoa at baseline was significantly higher for girls than boys (odds ratio (OR) = 1.97; 95% CI 1.32–2.93) (Table 2). There was a trend (*p* <0.1) that girls were at a higher odds of being infected with *E. coli* than boys (OR = 1.40; 95% CI 1.00–1.96). Children from Wawi school had significantly lower odds of being infected with *B. hominis* (OR = 0.64; 95% CI 0.43-0.97), *E. nana* (OR = 0.63; 95% CI 0.40–1.00) and *I. bütschlii* (OR = 0.50; 95% CI 0.25–1.00) considering only the baseline stool sample than children from Al-Sadik. Additionally, there was a trend (*p* <0.1) that children from Wawi school had a lower odds of being infected with *G. intestinalis* (OR = 0.66; 95% CI 0.41–1.07) and any of the three (potentially) pathogenic intestinal protozoa combined (OR = 0.68; 95% CI 0.47–1.00).

**Table 2:** Odds ratios (OR) of being infected with intestinal protozoa among school-aged children on Pemba Island in mid-2011, as assessed by logistic regression

**Intestinal protozoa**	**OR girls vs. boys (95% CI)**	**OR Wawi vs. Al-Sadik school (95% CI)**
*G. intestinalis*	0.67 (0.44–1.11)	0.66 (0.41–1.07)
*E. histolytica/E. dispar*	1.21 (0.78–1.87)	1.30 (0.77–2.19)
*B. hominis*	0.94 (0.64–1.37)	0.64 (0.43–0.97)*
*E. coli*	1.40 (1.00–1.96)	1.04 (0.71–1.53)
*E. nana*	1.34 (0.87–2.05)	0.63 (0.40–1.00)*
*E. hartmanni*	1.15 (0.70–1.89)	0.87 (0.50–1.50)
*C. mesnili*	1.65 (0.88–3.09)	0.74 (0.38–1.44)
*I. bütschlii*	1.04 (0.53–2.04)	0.50 (0.25–1.00)*
*G. intestinalis* or *E. histolytica/E. dispar* or *B. hominis*	1.13 (0.81–1.58)	0.68 (0.47–1.00)
Any intestinal protozoa	1.97 (1.32–2.93)*	0.72 (0.46–1.14)

*p <0.05.

CI, confidence interval.

### Effect of antiparasitic treatment against intestinal protozoa

Observed CRs were moderate to high for all intestinal protozoa regardless of the treatments administered (Table 3). The highest CR was observed for the albendazole-nitazoxanide combination against *E. nana* (CR 91.3%; 95% CI 78.8–100.0%)*.* The lowest CR among the antiparasitics tested was documented for single-dose albendazole against *E. coli* (CR 33.3%; 95% CI 22.4–44.3%). Note that the group of children receiving placebo had moderate to high CRs against intestinal protozoa infections. Comparing the outcomes among treatment arms using logistic regression revealed that single-dose nitazoxanide had a significant effect on *E. coli* (OR = 0.35; 95% CI 0.18–0.68). Furthermore, we observed a trend (*p* <0.1) with the combination of nitazoxanide plus albendazole against *E. nana* (OR = 0.18; 95% CI 0.02–1.27). All other results of the logistic regressions revealed p-values above 0.1; hence, there was no significant effect compared to placebo.

**Table 3:** Effect of albendazole, nitazoxanide, sequentially administered albendazole-nitazoxanide combination, and placebo against intestinal protozoa infections among school-aged children on Pemba Island in mid-2011

**Characteristic**	**Single-dose albendazole**	**Single-dose nitazoxanide**	**Nitazoxanide-albendazole combination**	**Placebo**
***G. intestinalis***				
No. of infected children	25	21	19	25
No. of children not cured after treatment	10	9	11	12
CR, % (95% CI)	60.0	57.1	42.1	52.0
	(39.4–80.6)	(34.1–80.2)	(17.7–66.6)	(31.0–73.0)
***E. histolytica/E. dispar***				
No. of infected children	23	23	31	22
No. of children not cured after treatment	10	9	10	10
CR, % (95% CI)	56.5	60.9	67.7	54.5
	(34.6–78.4)	(39.3–82.4)	(50.3–85.2)	(31.9–77.1)
***B. hominis***				
No. of infected children	35	37	33	49
No. of children not cured after treatment	15	16	10	16
CR, % (95% CI)	57.1	56.8	69.7	67.3
	(39.9–74.4)	(40.0–73.5)	(53.1–86.2)	(53.7–81.0)
***E. coli***				
No. of infected children	75	82	56	70
No. of children not cured after treatment	50	38	27	50
CR, % (95% CI)	33.3	53.7	51.8	28.6
	(22.4–44.3)	(42.6–64.7)	(38.3–65.3)	(17.7–39.4)
***E. hartmanni***				
No. of infected children	14	22	18	19
No. of children not cured after treatment	5	5	4	7
CR, % (95% CI)	64.3	77.3	77.8	63.2
	(35.6–93.0)	(58.3–96.3)	(56.5–99.1)	(39.3–87.0)
***E. nana***				
No. of infected children	24	25	23	35
No. of children not cured after treatment	10	9	2	15
CR, % (95% CI)	58.3	64.0	91.3	57.1
	(37.1–79.6)	(43.8–84.2)	(78.8–100.0)	(39.9–74.4)
***C. mesnili***				
No. of infected children	17	10	6	12
No. of children not cured after treatment	4	1	1	5
CR, % (95% CI)	76.5	90.0	83.3	58.3
	(54.0–99.0)	(67.4–100.0)	(40.5–100.0)	(25.6–91.1)
***I. bütschlii***				
No. of infected children	5	13	8	11
No. of children not cured after treatment	1	2	2	4
CR, % (95% CI)	80.0	84.6	75.0	63.6
	(24.5–100.0)	(61.9–100.0)	(36.3–100.0)	(29.7–97.5)

CI, confidence interval; CR, cure rate.

Comparing the mean intensity of intestinal protozoa infection before and 3 weeks after treatment in the different arms revealed no significant effect for most of the assessed intestinal protozoa (results not shown). The only significant reductions of infection intensity (95% CI below 0) were observed in the albendazole-nitazoxanide combination against *E. histolytica/E. dispar* (−0.21; 95% CI -0.34 to -0.09) and *E. coli* (−0.37; 95% CI -0.58 to -0.16) and in the nitazoxanide single-dose treatment against *E. coli* infection intensity (−0.37; 95% CI -0.58 to -0.16)*.*

## Discussion

Since very little is known on the epidemiology of intestinal protozoa in sub-Saharan Africa, we analyzed formalin-fixed stool samples obtained from 550 school-aged children who participated in a randomized controlled trial on Pemba Island to assess the efficacy and safety of nitazoxanide, albendazole and a combination of both drugs against *T. trichiura* and other soil-transmitted helminths. This trial found low CRs against soil-transmitted helminth species [19]. Importantly, the study provided an opportunity to shed new light on the extent of intestinal protozoa infections in a child cohort and to determine whether the different study treatments had an effect on intestinal protozoa species.

Our results confirm that intestinal protozoa are a public health issue on Pemba Island. Indeed, almost half of the children were infected with at least one of the three (potentially) pathogenic intestinal protozoa. When considering the results from the baseline and the 3-week post-treatment follow-up, assuming that a child who was diagnosed positive at follow-up was diagnosed as a false-negative case at baseline, the prevalence of intestinal protozoa infection was even higher (Figure 1). This issue is most likely explained by the lack of sensitivity when examining only a single stool sample with the ether-concentration technique, an important limitation of our study. Hence, in future studies, multiple stool samples should be examined and subjected to the ether-concentration method or more sensitive molecular approaches employed to improve diagnostic accuracy [26,27].

A recent study reported only moderate sensitivity for the ether-concentration technique compared to the FLOTAC technique when examining the same formalin-fixed stool samples [28]. However, a study among five European reference laboratories showed that the agreement of diagnostic results was only moderate for pathogenic intestinal protozoa although the participating centres adhered to the same standard operating procedures for the ether-concentration technique [21]. In particular, *E. histolytica* is frequently misdiagnosed, even by experienced laboratory personnel [29].

We found that girls were generally at higher odds of an infection with any of the intestinal protozoa encountered than boys. Similar results were found by Mohammed Mahady and colleagues in Malaysia [30]. On the other hand, Traoré *et al.*, in a study carried out in school-aged children in Côte d’Ivoire, reported a considerably higher prevalence of intestinal protozoa among boys than girls [31], corroborating findings by Cifuentes *et al.* from Mexico, where boys were at higher odds of a *G. intestinalis* infection that girls [32]. Other studies found no gender difference at all [33]. These findings indicate that intestinal protozoa infections may be related to gender-specific behaviour within a community. In addition, we observed statistically significant differences for some of the intestinal protozoa between the two schools. Hence, even though the schools are located only a few kilometers apart and the two settings were quite similar, at least in terms of socioeconomic status, availability of safe water supply and sanitation infrastructure, different infection profiles were observed. This highlights the possibility of ‘micro-geographic’ variability in endemicity of intestinal protozoa infection. One explanation could be that Al-Sadik school is located close to an orphanage, where transmission of intestinal protozoa might be enhanced. Other behavioural, infrastructure or environmental factors that may account for our observation should be investigated in future studies.

Intestinal protozoa often co-occur with intestinal nematodes and it is therefore important to determine whether anthelminthic and other antiparasitic drugs have an effect on concomitant intestinal protozoa infections. Albendazole, for example, was found in a recent meta-analysis to be as effective as metronidazole against *G. intestinalis*[34]. The observed CRs against all intestinal protozoa were moderate to high in the three treatment groups. However – and contrary to what we expected – there was also a moderate treatment efficacy in the placebo group, which is difficult to explain other than a diagnostic dilemma. Our findings therefore have to be interpreted with caution. The high ‘cure rate’ observed within the placebo group underscores that analysis of a single stool sample with the formalin-ether concentration technique is unreliable, and hence, the CRs for the three treatment schemes investigated here are likely overestimated. Still, our results show that none of the drugs administered as single dose resulted in cure of all infected children. Only moderate CRs were observed against *G. intestinalis*, *E. histolytica/E. dispar* and the potentially pathogenic *B. hominis.* A further limitation is the relatively small number of positive children for specific intestinal protozoa. Infection intensity did not significantly decrease after treatment with the exception of *E. coli* in the nitazoxanide group. Note that intestinal protozoa multiply within the host, which can also influence infection intensity results [35]. Further, the incubation time for different intestinal protozoa is between 7 and 28 days, meaning that it is possible that within the 3-week period before and after treatment, re-infection had occured [36-40]. The clinical relevance of the moderate CRs suggests that single-dose albendazole or nitazoxanide or a combination of the two drugs do not have sufficient efficacy against pathogenic intestinal protozoa. However, multiple stool samples should be examined to strengthen the diagnostic accuracy for these infections.

## Conclusion

Our study revealed that intestinal protozoa infections are highly prevalent among school-aged children on Pemba Island, a setting where these intestinal protozoa were not considered of major importance thus far. The difficulty in accurately diagnosing intestinal protozoa infections, also experienced in our study, might be one of the chief reasons why these pathogens and the diseases they cause are often neglected, even in settings where mass treatment are underway against intestinal helminth infections [21]. The prevalence of intestinal protozoa is therefore unknown in many settings and consequently the disease burden cannot be fully quantified [41]. Accurate diagnostic tools that can be applied at the point-of-care are thus urgently required. Finally, drugs and effective treatment regimens for intestinal protozoa infections to be used in public health campaigns are still far out of reach.

## Competing interests

None of the authors has any conflict of interest concerning the work reported in this paper.

## Authors’ contributions

BS, ShMA, SaMA, JU, MA and JK designed the study; BS, HM, ShMA, SaMA, and JK implemented the study; BS managed the data; BS, HM, IIB, JU and JK analyzed and interpreted the data; BS wrote the first draft of the paper; HM, ShMA, SaMA, IIB, JU, MA and JK revised the manuscript. All authors read, and approved the manuscript prior to submission and assisted with the final revision of the manuscript.
